# A Prognostic Model for Glioblastoma Patients Treated With Standard Therapy Based on a Prospective Cohort of Consecutive Non-Selected Patients From a Single Institution

**DOI:** 10.3389/fonc.2021.597587

**Published:** 2021-02-25

**Authors:** Armita Armina Abedi, Kirsten Grunnet, Ib Jarle Christensen, Signe Regner Michaelsen, Aida Muhic, Søren Møller, Benedikte Hasselbalch, Hans Skovgaard Poulsen, Thomas Urup

**Affiliations:** ^1^Department of Radiation Biology, The Finsen Center, Rigshospitalet, Copenhagen, Denmark; ^2^Department of Oncology, The Finsen Center, Rigshospitalet, Copenhagen, Denmark; ^3^Department of Gastroenterology, Hvidovre Hospital, Hvidovre, Denmark; ^4^Biotech, Research & Innovation Centre (BRIC), University of Copenhagen, Copenhagen, Denmark

**Keywords:** glioma grade IV, nomogram, prognostic factors, overall survival, progression-free survival, glioblastoma, biomarkers, MGMT = O^6^-DNA-methylguanine methyltransferase

## Abstract

**Background:**

Glioblastoma patients administered standard therapies, comprising maximal surgical resection, radiation therapy with concomitant and adjuvant temozolomide, have a variable prognosis with a median overall survival of 15–16 months and a 2-year overall survival of 30%. The aim of this study was to develop a prognostic nomogram for overall survival for glioblastoma patients treated with standard therapy outside clinical trials.

**Methods:**

The study included 680 consecutive, non-selected glioblastoma patients administered standard therapy as primary treatment between the years 2005 and 2016 at Rigshospitalet, Copenhagen, Denmark. The prognostic model was generated employing multivariate Cox regression analysis modeling overall survival.

**Results:**

The following poor prognostic factors were included in the final prognostic model for overall survival: Age (10-year increase: HR = 1.18, 95% CI: 1.08–1.28, p < 0.001), ECOG performance status (PS) 1 vs. 0 (HR = 1.30, 95% CI: 1.07–1.57, p = 0.007), PS 2 vs. 0 (HR = 2.99, 95% CI: 1.99–4.50, p < 0.001), corticosteroid use (HR = 1.42, 95% CI: 1.18–1.70, p < 0.001), multifocal disease (HR = 1.63, 95% CI: 1.25–2.13, p < 0.001), biopsy vs. resection (HR = 1.35, 95% CI: 1.04–1.72, p = 0.02), un-methylated promoter of the MGMT (O^6^-methylguanine-DNA methyltransferase) gene (HR = 1.71, 95% CI: 1.42–2.04, p < 0.001). The model was validated internally and had a concordance index of 0.65.

**Conclusion:**

A nomogram for overall survival was established. This model can be used for risk stratification and treatment planning, as well as improve enrollment criteria for clinical trials.

## Introduction

Glioblastoma, defined as WHO grade IV astrocytoma, is the most frequent and lethal primary brain tumor in adults (1). The standard therapy of newly diagnosed glioblastoma includes maximal surgical resection followed by radiation therapy, 60 Gy in 30 fractions, 5F/W plus concomitant and six series of adjuvant temozolomide (2). Despite this aggressive treatment the prognosis is poor with a median overall survival of 15–16 months (2–4). However, the survival of an individual patient varies greatly, with some patients experiencing tumor progression shortly after initiation of treatment, whereas approximately 30% of patients are alive after 2 years (2, 5). Therefore, it is of importance to establish prognostic models that can be used prior to initiation of concomitant treatment for risk stratification and treatment planning as well as to improve enrollment criteria for clinical trials.

In glioblastoma patients treated with standard therapy outside clinical trials, the underlying clinical and molecular factors for distinguishing short-term survivors from long-term survivors remain poorly understood. Two molecular factors, the promoter methylation of the O^6^-methylguanine-DNA methyltransferase (MGMT) gene and the mutation of the isocitrate dehydrogenase-1 (IDH-1), an indicator of progression from lower grade glioma (secondary glioblastoma), have been identified as prognostic factors associated with a favorable prognosis (6–8). The most consistent and well-described clinical prognostic factors associated with a poor survival include: increasing age, poor performance status (PS), low degree of surgical resection of tumor, and the use of corticosteroids (4, 9–11).

We have previously from a cohort of 225 glioblastoma patients, established a prognostic model on the basis of three independent poor prognostic factors, namely increasing age, poor performance status, and corticosteroid use at treatment start (12). The aim of this study is to develop a refined prognostic model in an expanded cohort of 680 prospectively included community based newly diagnosed glioblastoma patients treated with standard therapy outside clinical trials.

## Material and Methods

### Patient Population

All glioblastoma patients, whom were administered standard treatment at Rigshospitalet, Denmark between February 2005 and December 2016, were prospectively registered in a database and included in this retrospective study. The study cut-off day was March 22^nd^ 2019. The study included 680 consecutive, non-selected glioblastoma patients of whom 225 glioblastoma patients (treated from 2005 to 2010) have been included in a previous study (12). The study was performed according to the Declaration of Helsinki and Danish legislation and was approved by the local ethical committee (H-19054690). Eligible patients for standard treatment had histologically verified glioblastoma, a performance status of ≤2 and adequate hematologic- (neutrocytes ≥1,500/mm^3^; thrombocytes ≥100,000/mm^3^), renal- (serum creatinine level, ≤1.5 times normal upper limit), and hepatic function (bilirubin level, ≤1.5 times the normal upper limit and liver enzymes <3 times the normal upper limit).

### Biomarker Analysis

Histological and immunohistochemical (IHC) analysis was performed as described in our previous paper by Michaelsen et al (12). IHC were immunostained on a DAKO Cytomation autostainer using murine monoclonal antihuman antibodies against ATRX (HPA001906, Sigma) and MGMT (MAB16200, 1:200, Millipore). IDH1 was either examined by IHC using anti-IDH1 R132H antibody (clone H09, Dianova, 1:700 dilution) or by Multiplex Ligation-dependent Probe Amplification (MLPA), using the SALSA MLPA kit P088 (MRC Holland). IDH1 and ATRX IHC staining were categorized as positive (mutated *IDH1*) or negative (mutated *ATRX*). Previous lower grade glioma or glioma harboring *IDH1* mutations were categorized as secondary glioblastomas. MGMT was categorized negative if the number of cells stained was less than 10%, while ≥10% was considered positive (12). From year 2014, MGMT status was evaluated by examining MGMT promoter methylation on purified DNA from the diagnostic sample by pyrosequencing using the Therascreen MGMT Pyro kit (Qiagen) and following the instructions of the manufacturer. Samples with mean methylation above a cut-off 10% were considered positive. We have previously shown that methylation of the *MGMT* promoter gene is inversely, highly and significantly correlated to expression by IHC (13), and MGMT status was classified based on IHC in 367 cases, which were not evaluated by pyrosequencing.

### Treatment and Follow-Up

All patients were evaluated for surgery at initial diagnosis. Subsequently, all patients were treated with radiation therapy (60 Gy/30 fractions, 5 fractions per week) with concomitant Temozolomide (75 mg per m^2^/day, 7 days per week) from the first day of radiation therapy until the last day of radiation therapy and intended six series of adjuvant temozolomide (150 mg/m^2^ for the first cycle and 200 mg/m^2^ in the following cycles) as described by Stupp et al. (2).

According to the Danish Neuro-Oncology Group guidelines, all patients were post-operatively provided with a plan for corticosteroid tapering. The tapering schedules include a gradual dose reduction over a period of 2–4 weeks to prevent rebound symptoms with longer periods for symptomatic patients. Patients that were still dependent on prednisolone > 10 mg/day at start of concomitant treatment, were defined as corticosteroid users.

Contrast and non-contrast magnetic resonance imaging (MRI) scans were performed after surgery and after 2- and 5, series of adjuvant temozolomide. Degree of resection at primary surgery was evaluated non-standardized by a neurosurgeon or standardized by a postoperative MRI scan performed within 72 h after resection, when available (14). Multifocal glioblastoma was defined by MRI as at least two non-connected contrast-enhancing tumors (not connected by T2/FLAIR signal) with foci at least 1 cm apart.

All patients were evaluated by the multidisciplinary team at Rigshospitalet. Treatment response was evaluated by a neuroradiologist according to the Macdonald criteria (15) together with clinical response evaluated by a clinical oncologist, and defined as either response (complete or partial response), stable disease or progressive disease.

All patients were subsequently followed and evaluated every 3 months until death or termination of follow up at our institution using the same procedures as described above.

At time of recurrence, patients who maintained performance status ≤2 were considered for salvage tumor resection and/or second line treatment with either reinduction of temozolomide (for late recurrences ≥6 months after completion of Stupp regimen), bevacizumab in combination with either lomustine or irinotecan (16, 17) or inclusion in a clinical trial.

### Statistical Analysis

Progression-free survival was defined as time from diagnosis to either progressive disease or death of any cause and overall survival was defined as time from diagnosis to death of any cause. Survival probabilities were estimated with the Kaplan-Meier method. Response was estimated by penalized maximum likelihood (modeling the probability of response) and the results were presented by odds ratios (OR) with 95% confidence intervals (95% CI). The Cox proportional hazards model was used for modeling survival endpoints and results are presented as hazard ratios (HR) with 95% CI. Assessment of the model assumptions was done using martingale residuals. Factors associated with response, progression-free survival overall survival with P-values below 0.10 in univariate analysis were considered for multivariate analysis. Multivariate analyses included complete cases; incomplete cases with missing data were excluded from the analysis. Some covariates have substantial missing values and therefore supplemental analyses have been included imputing missing values based on 25 imputations (18). The estimated survival probabilities for 6, 12, and 18 months have been estimated based on the Cox regression model. The concordance index (C-index) was calculated as a measure of discrimination (19). Ten-fold cross-validation was applied to the analysis of overall survival in order to assess the estimated model. The significance level was set to 5%. All statistical calculations have been made using SPSS, SAS (v9.4, SAS Institute, Cary, N.C., USA) and R (v 3.1.0 R Development Core team, Vienna, Austria, http://www.R-project.org) (package RMS).

## Results

### Characteristics of Patient Cohort

A total of 680 glioblastoma patients treated with standard therapy were included in the study (65% male) with a median age of 60 years (range: 17–79). Most patients (85%) underwent initial surgery, by surgically defined complete resection (62%) or partial resection (38%), while the remaining 101 (15%) patients underwent a diagnostic biopsy due to non-resectability (**Table 1**). Extent of resection by post-operative MRI was assessed in 354 patients (52%) of whom 130 (37) had complete resection and 222 (63%) had partial resection. At the initiation of concomitant radiation-chemotherapy, most patients were in good ECOG performance status (PS 0–1, 93%) and 39% of patients were tapered off corticosteroids. The majority of patients had unifocal disease (88%), while the remaining patients (12%) had multifocal disease. Of 564 patients analyzed for MGMT, 300 patients (53%) were MGMT methylated and the remaining 47% were unmethylated. Secondary glioblastomas (defined as harboring IDH1 mutation or previous lower grade glioma) included 35 patients (5%). The patients received a median of four cycles of adjuvant temozolomide (range: 0–12) and 17 patients received more than six cycles of adjuvant temozolomide.

**Table 1:** Patient characteristics (*n* = 680).

**Age (years), median (range)**	60.2 (17.1–79.6)
**Gender, *n* (%)**	
Female	238 (35)
Male	442 (65)
**ECOG performance status, *n* (%)**	
0	415 (62)
1	220 (33)
2	37 (5)
Missing	8
**Tumor size, median (range)**	16 cm2 (0.25–61.5)
Missing	345
**Multifocal Disease, *n* (%)**	
Yes	83 (12)
No	595 (88)
Missing	2
**Secondary glioblastoma****^a^****, *n* (%)**	
Yes	35 (5)
No	645 (95)
Missing	0
**MGMT status, *n* (%)**	
Methylated	300 (53)
Unmethylated	264 (47)
Missing	116
**ATRX status, *n* (%)**	
Positive	135 (91)
Negative	13 (9)
Missing	532
**EGFR status, *n* (%)**	
Positive	440 (82)
Negative	95 (18)
Missing	145
**Tumor resection, *n* (%)**	
Biopsy	101 (15)
Resection	579 (85)
**Surgically defined extent of resection, *n* (%)**	
Partial resection	254 (38)
Complete resection	95 (62)
Missing	7
**MRI defined extent of resection****^b^****, *n* (%)**	
Partial resection	222 (63)
Complete resection	130 (37)
Missing	227
**Corticosteroid use****^c^****, *n* (%)**	
Yes	412 (61)
No	262 (39)
Missing	6
**No. of adjuvant Temozolomide, *n* (%)**	
Median	4
Range	0–12
>6 cycles	17 (2)
**Best clinical response, *n* (%)**	
Response (Complete response + partial response)	57 (9)
Stable disease	320 (49)
Progressive disease	271 (42)
Missing	32
**Follow-up duration (months), median (range)**	80 (25.8–169.4)

MGMT, O^6^-methylguanine-DNA methyltransferase; ATRX, alpha thalassemia/mental retardation syndrome X-linked; EGFR, epidermal growth factor; MRI, magnetic resonance imaging.

a Prior diagnosis with lower grade glioma or glioblastoma with IDH1 mutation.

b Assessed by MRI within 72 h after surgery.

c Prednisolone dose > 10 mg/day at initiation of concomitant treatment.

At first recurrence, 250 patients (38%) underwent surgical resection and 2^nd^ line treatment was administered in 363 patients (53%). As shown in **Table 2**, re-induction with temozolomide was administered in 70 patients at first recurrence. At first or later recurrence 192 patients received bevacizumab plus irinotecan, 105 patients bevacizumab plus lomustine, 24 patients lomustine alone and 2 patients bevacizumab alone. Fifty-three patients were administered various experimental treatments in phase-2 trials and 22 patients were treated with re-irradiation therapy (20, 21). At time of analysis, 32 (5%) patients were alive out of which 20 patients showed no progression and 646 (95%) patients had deceased. The median follow-up time from the date of treatment initiation was 80 months (Range: 26–169). Further patient characteristics are presented in **Supplementary Table 1**.

**Table 2:** Pattern of progression and relapse treatment (*n* = 660).

**Pattern of relapse**	
Local	416 (77)
Multifocal	125 (23)
Missing^a^	119
**Reoperation, *n* (%)**	
Yes	250 (38)
No	410 (62)
**Temozolomide, *n* (%)**	
Yes	70 (11)
No	590 (89)
**Bevacizumab plus chemotherapy****^a^****, *n* (%)**	
Yes	290 (44)
No	370 (56)
**Combined reoperation and bevacizumab plus chemotherapy****^a^****, *n* (%)**	
Yes	144 (22)
No	516 (78)

a Irinotecan or lomustine.

### Treatment Outcome in Relation to Diagnostic Year

**Figure 1:**
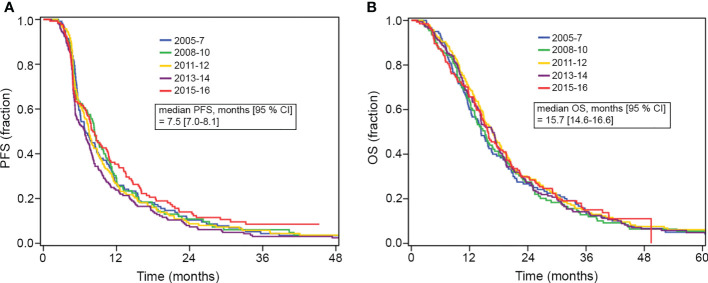
**(A, B)** Kaplan-Meier plots of progression-free survival (PFS) and overall survival (OS) for patients grouped according to year of diagnosis (2-year interval). Median PFS and OS are shown for the total cohort.

The median progression-free survival was 7.5 months (95% CI: 7.0–8.1) and the median overall survival was 15.7 months (95% CI: 14.6–16.6). In order to investigate whether the survival outcome had changed over the years; we categorized the patients according to diagnostic years in a 2-year interval and analyzed progression-free survival and overall survival according to each group. As shown in **Figures 1A, B**, no significant difference in progression-free survival (p = 0.19) and overall survival (p = 0.95) was observed over the years.

### Factors Associated With Response, Progression-Free Survival and Overall Survival, Univariate Analysis

All factors shown in **Supplementary Table 1** were screened for association with best response, progression-free survival and overall survival by univariate analysis (**Supplementary Table 2**).

As shown in **Table 1**, best responses to standard treatment among evaluable patients were: response in 57 patients (9%), stable disease in 320 patients (49%), and progressive disease in 271 patients (42%). In this study, all patients were assessed response evaluable, including patients having complete surgical resection. This may explain why smaller tumor size was associated with poor response (p = 0.057). Other factors found associated with a poor response were: Un-methylated MGMT (p = 0.01), poor performance status (p = 0.02), use of corticosteroids (p = 0.09), biopsy compared to resection (p = 0.054), biopsy compared to surgically defined extent of resection (p = 0.059), and higher age (p = 0.007).

In univariate analysis, factors associated with poor progression-free survival were: Poor performance status (p < 0.001), higher age (p < 0.001), corticosteroid use at start of concomitant treatment (p < 0.001), un-methylated MGMT (p<0.001), secondary glioblastoma (p < 0.001), multifocal disease (p < 0.001), biopsy compared to resection (p < 0.001), and extent of resection defined by post-operative MRI (p = 0.002) and by the surgeon (p < 0.001).

Factors associated with poor overall survival by univariate analysis were: Poor performance status (p < 0.001), higher age (p < 0.001), corticosteroid use (p < 0.001), un-methylated MGMT (p < 0.001), multifocal disease (p < 0.001), biopsy compared to resection (p < 0.001), extent of resection defined by post-operative MRI (p = 0.001) and by the surgeon (p < 0.001), and secondary glioblastoma (p = 0.004). For patients with secondary glioblastoma, the median overall survival was 28.1 months (95% CI 16.7–39.6) and for patients with primary glioblastoma the median overall survival was 15.2 months (95% CI 14.3–16.1).

### Multivariate Analysis of Response

**Table 3** summarizes the multivariate analyses for response and survival endpoints. By multivariate analysis two prognostic factors were associated with a poor likelihood of achieving response: multifocal disease (OR = 0.57, 95% CI: 0.33–0.99, p = 0.05) and un-methylated MGMT (OR = 0.67, 95% CI: 0.51–0.87, p = 0.003). Performance status, tumor size, corticosteroid use, biopsy compared to resection, extent of resection (post-operative MRI and surgically defined resection), and age were not significantly associated with response when added to the model. Nevertheless, it was decided to adjust the model for all independent prognostic factors associated with overall survival. The concordance index (C-index) for the final response model was 0.67 which is interpreted into a 67% probability of agreement between predicted and actual observed response (22).

**Table 3:** Multivariate analysis: response, progression-free survival and overall survival.

	Response OR [95% CI]	Progression-free survival HR [95% CI]	Overall Survival HR [95% CI]
Age, per 10-year increase	0.81 [0.64–1.02]	1.06 [0.98–1.15]	1.18 [1.08–1.28]
	p = 0.07	p = 0.2	p < 0.001
MGMT status, un-methylated vs. methylated	0.67 [0.51–0.87]	1.38 [1.15–1.65]	1.71 [1.42–2.04]
	p = 0.003	p < 0.001	p < 0.001
Corticosteroid use, yes vs. no	1.15 [0.88–1.51]	1.22 [1.01–1.47]	1.42 [1.18–1.70]
	p = 0.3	p = 0.04	p < 0.001
Multifocal, yes vs. no	0.57 [0.33–0.99]	1.62 [1.23–2.12]	1.63 [1.25–2.13]
	p = 0.05	p < 0.001	p < 0.001
ECOG PS 1 vs. 0	0.79 [0.50–1.23]	1.24 [1.02–1.51]	1.30 [1.07–1.57]
	p = 0.3	p = 0.03	p = 0.007
ECOG PS 2 vs. 0	1.71 [0.89–3.27]	1.97 [1.29–3.01]	2.99 [1.99–4.50]
	p = 0.1	p = 0.002	p < 0.001
Biopsy vs. resection	0.88 [0.60–1.30)]	1.37 [1.05–1.75]	1.35 [1.04–1.72]
	p = 0.5	p = 0.02	p = 0.02

Only complete cases were included; incomplete cases with missing data were excluded from the analysis. Missing data: 128 cases.

MGMT, O^6^-methylguanine-DNA methyltransferase; PS, performance status.

#### Multivariate Analysis of Progression-Free Survival

Multivariate analysis of progression-free survival found five independent factors associated with a higher risk of progression or death (**Table 3**): Un-methylated MGMT (HR = 1.38, 95% CI: 1.15–1.65, p < 0.001), corticosteroid use (HR = 1.22, 95% CI: 1.01–1.47, p = 0.04), multifocal disease (HR = 1.62, 95% CI: 1.23–2.12, p < 0.001), ECOG performance status 1 vs. 0 (HR = 1.24, 95% CI: 1.02–1.51, p = 0.03), ECOG performance status 2 vs. 0 (HR = 1.97, 95% CI: 1.29–3.01, p = 0.002), and biopsy vs. resection (HR = 1.37 95% CI: 1.05–1.75, p = 0.02). By replacing the latter, surgically defined extent of resection was not associated with progression-free survival (p = 0.6). In our study, extent of resection defined by post-operative MRI was not associated with progression-free survival (p = 0.2). When added to the model, secondary glioblastoma was independently associated with improved progression-free survival (HR 0.52, 95%CI: 0.34–0.78, p = 0.002). It was chosen not to include secondary glioblastoma due to the low frequency of cases. Age was not independently associated with progression-free survival (p = 0.2). However, it was decided to keep age in the model because age was an independent prognostic factor associated with overall survival. The C-index for the progression-free survival model was 0.60.

### Multivariate Analysis of Overall Survival

Of the factors identified significant by univariate analysis, six were found significantly associated with poor overall survival by multivariate analysis and were included in the final predictor (**Table 3**): Increasing age (HR = 1.18, 95% CI: 1.08–1.28, p < 0.001), un-methylated MGMT (HR = 1.71, 95% CI: 1.42–2.04, p < 0.001), corticosteroid use (HR = 1.42, 95% CI: 1.18–1.70, p < 0.001), multifocal disease (HR = 1.63, 95% CI: 1.25–2.13, p < 0.001), EGOC performance status 1 vs. 0 (HR = 1.30, 95% CI: 1.07–1.57, p < 0.007), EGOC performance status 2 vs. 0 (HR = 2.99, 95% CI: 1.99–4.50, p < 0.001), and biopsy vs. resection (HR = 1.35 95% CI:1.04–1.72, p = 0.02). Extents of resection defined by the surgeon (p = 0.055) and by post-operative MRI (p = 0.4) were not significantly associated with overall survival. Secondary glioblastoma was independently associated with improved overall survival (p = 0.004) but was not included in the final model due a low frequency of cases. The C-index for the final overall survival model was 0.65.

### Evaluation of the Prognostic Model for Overall Survival

The final prognostic model for overall survival underwent internal cross validation which confirmed the estimated model. In this validation analysis, all six covariates were significant in all cases. Because of incomplete cases, especially due to 116 patients with lack of MGMT-status, multiple imputation was performed to simulate missing values of the covariates. This analysis, shown in **Supplementary Table 3**, did not change the HR of the covariates and all covariates were significant, except for multifocal disease which was borderline significant (p = 0.053) and biopsy vs. resection which showed no association (p = 0.13). Accordingly, incomplete cases did not impact the prognostic model profoundly but influenced the more infrequent factors such as biopsy and multifocal disease. Accordingly, the final prognostic model for overall survival was validated successfully and showed a high degree of robustness by multiple imputation analysis.

Applying the final prognostic model, shown in **Table 3**, overall survival can be estimated using the prognostic nomogram able to calculate the hazard score based on the estimated vector from the Cox regression (XB):

$$XB=0.164\times \frac{Age}{10}+0.261\times (PS=1)+1.096\times (PS=2)+0.348\times Steroid+0.490\times Multifocal+0.297\times Biopsy+0.534\;*\;MGMT(unmethylated)$$

**Figure 2:**
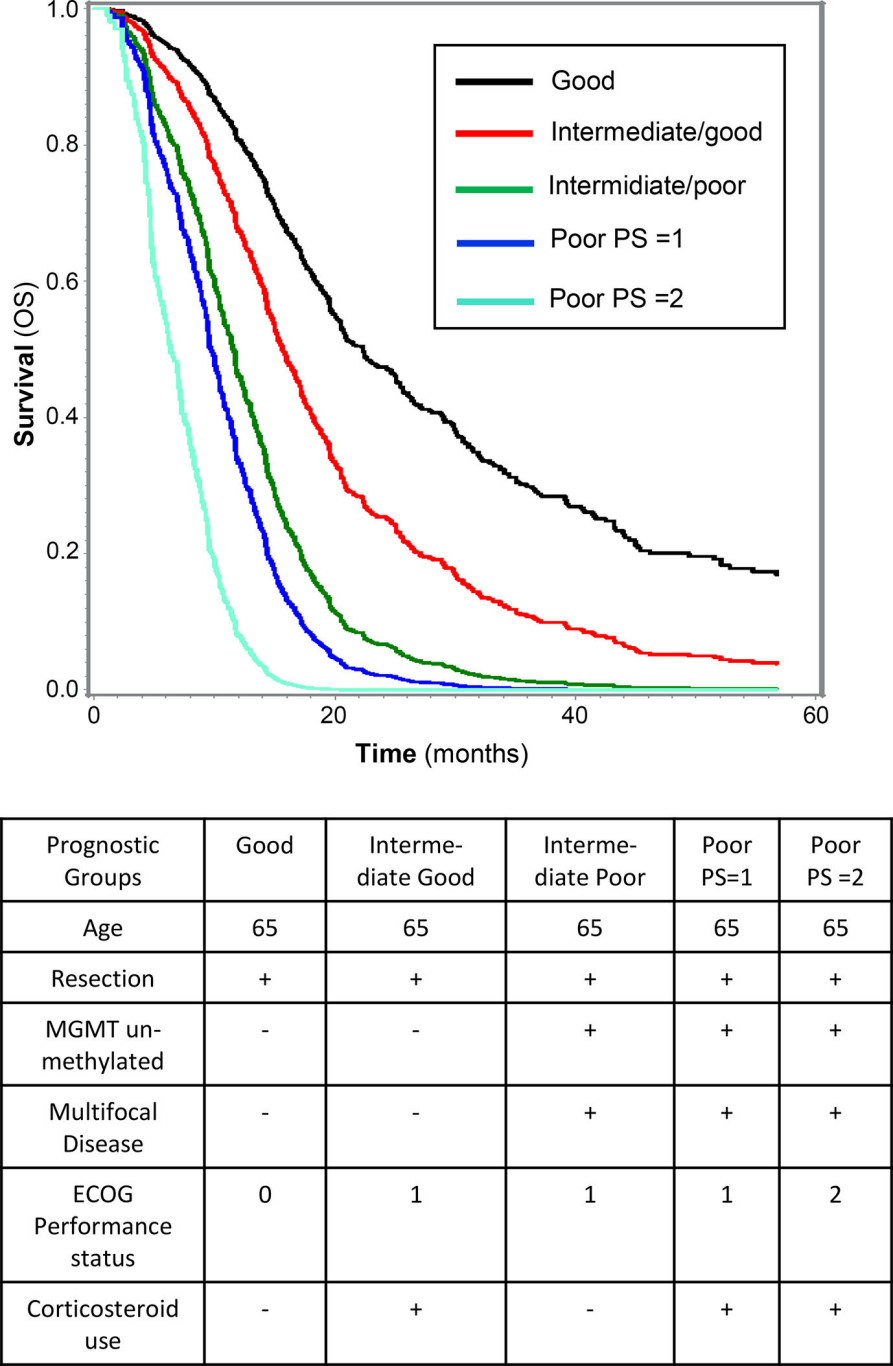
Survival of five prognostic groups, illustrated by the estimated survival curves based on combinations of the six independent prognostic factors included in the final model for overall survival. For all illustrated prognostic groups, defined in the table, the age is 65 years, and all have had undergone resection.

As an example, shown in **Figure 2**, the estimated survival curves of four prognostic groups represented by a fixed age (65 years) and all having undergone resection: For the best prognostic group (called Good) characterized by PS = 0, no use of corticosteroids, unifocal disease, and a MGMT-methylated tumor the estimated median overall survival is 22.3 months (95% CI: 19.4–25.7). In contrast, patients in the poor prognostic group (called Poor PS = 2) represented by PS = 2, use of corticosteroids, un-methylated MGMT, and multifocal disease the estimated median overall survival is 7.8 months (95% CI: 5.0–9.5).

Furthermore, a prognostic index was established using the hazard score (XB). Thresholds for overall survival at 12, 18, and 24 months were set to 50% and XB according to each threshold was used to define four prognostic groups: Poor (XB > 1.98), Intermediate poor (XB = 1.11–1.98), Intermediate good (XB = 0.69–1.11), and Good (XB < 0.69). The distribution of the study cohort according to the prognostic groups and overall survival is listed in **Table 4**. As an example, 75% of the patients in the Poor prognostic group had died within 12 months and 4% survived more than 24 months. In contrast, 4% of the patients in Good prognostic group died within the first year and 68% survived more than 24 months. It is more difficult to distinguish the groups Intermediate good and Intermediate poor. However, it is noticed that 44% survived more than 24 months in the Intermediate good group. In contrast, 21% survived more than 24 months in the Intermediate poor group. As an example, application of the prognostic nomogram to calculate the hazard score based on the estimated vector from the Cox regression (XB); a 60 year old patient with a partially resected tumor, using corticosteroids, ECOG PS = 1, unifocal disease, and unmethylated MGMT, would have a calculated hazard score of 2.13 and according to the established prognostic index, be classified as the prognostic group Poor (XB > 1.98).

**Table 4:** Distribution of prognostic groups and estimated overall survival.

Prognostic Groups	OS <12 months (%)	12 < OS <18 months (%)	18 < OS < 24 months (%)	OS >24 months (%)	Total
**Good** (XB^a^ < 0.69)	2 (4)	8 (16)	6 (12)	33 (68)	49
**Intermediate good** (XB = 0.69–1.11)	24 (19)	29 (22)	19 (14)	56 (44)	128
**Intermediate poor** (XB = 1.11–1.98)	104 (35)	86 (29)	45 (15)	64 (21)	299
**Poor** (XB > 1.98)	57 (75)	10 (13)	6 (8)	3 (4)	76

Only complete cases were included, incomplete cases with missing data were excluded from the analysis. Missing data: 128 cases.

a Hazard score (XB) calculated with application of the prognostic nomogram.

To summarize, we established a robust prognostic model able to estimate the probability of overall survival at different relevant timepoints.

## Discussion

In this study of 680 newly diagnosed glioblastoma patients treated with standard therapy, the objective was to develop a prognostic model for overall survival. Six independent prognostic factors were found associated with a poor survival. These were increasing age, poor PS, corticosteroid use, multifocal disease, un-methylated MGMT status, and biopsy versus resection. The six factors were included in a clinically relevant model that can predict the probability of survival.

The patients of the study were included consecutively and are therefore representative for the general population of newly diagnosed glioblastoma patients treated outside clinical trials. Although retrospectively evaluated, the observed survival outcome was comparable to previous prospective studies with a median progression-free survival and overall survival of 7.5 months and 15.7 months (2, 3, 23).

This study is a follow-up on our previous study by Michaelsen et al. in which 225 newly diagnosed glioblastoma patients were included to develop a prognostic model for overall survival (12). This model included three independent prognostic factors associated with early mortality, namely poor PS, corticosteroid use, and higher age. The current study analyzed an expanded cohort of patients and confirmed the prognostic model of our previous study. In addition, three additional independent prognostic factors were associated with poor overall survival: un-methylated MGMT, multifocal disease, and biopsy compared to resection. In contrast to MGMT-status and extent of resection which both are well-described prognostic factors (4, 10, 24), multifocal disease has not previously been identified as an independent prognostic factor in glioblastoma patients treated with standard therapy. Furthermore, our study found multifocal disease and un-methylated MGMT as being independently associated with a reduced likelihood of achieving response and poor progression-free survival, suggesting that these two factors may predict less benefit of standard therapy.

Currently available prognostic models established by Gorlia et al. and Gittleman et al. for newly diagnosed glioblastoma patients administered standard therapy, have been based on patients treated in clinical trials (4, 10). Consistently, these two studies include age, extent of resection, MGMT-status, and PS as predictors for overall survival, and inconsistently Mini-Mental State Examination (MMSE) and gender. In our study MMSE was not available and gender was not associated with survival. Extent of resection was not significantly associated with overall survival in our study. The prognostic model of this study was successfully validated internally and was to a high degree comparable to the model established by Gittleman et al. (4), which did not include corticosteroid use and multifocal disease. Accordingly, we suggest that our established model can be used in clinical practice to estimate the prognosis of glioblastoma patients treated with standard therapy. In line with the previous studies (4, 10), the established prognostic model had a C-index of 0.65 which means a 65% probability of agreement between predicted and actual observed survival, suggesting that the model can estimate survival with a reasonable precision. This however also suggests that there remain unknown explanatory factors associated with survival. Despite advances in histological and molecular subtypes, the factors included in prognostic models are mainly clinical observations such as PS, age and corticosteroid treatment (4, 9–12). Tissue biomarkers and data from imaging techniques have been suggested as independent prognostic factors (6, 25, 26), and may contribute to improvement of future prognostic models.

This study has some limitations. The single center study design is a limitation and our findings may or may not be generalizable to other centers. Accordingly, future studies could include validation of the nomogram in an independent cohort of consecutive patients treated at other centers. Another limitation is the relatively large number of incomplete cases for the multivariate analysis. However, multiple imputations were performed to adjust for possible selection bias and results from this analysis showed that incomplete cases had no significant impact on our final prognostic model.

Taken together, we established and validated a robust prognostic nomogram that included six independent prognostic factors: Age, PS, corticosteroid use, MGMT methylation status, multifocal disease, and degree of resection. The prognostic model can be a valuable tool for physicians to objectively inform patients about their prognosis and collaboratively decide which therapeutic modality is optimal for the individual glioblastoma patient. In our study we found no improvement in survival since the implementation of standard therapy in 2005. Therefore, improved knowledge regarding prognostic groups should be utilized in the design of future clinical trials in order to improve glioblastoma treatment.

## Data Availability Statement

The datasets for this article are not publicly available due to participant confidentiality. Requests to access the datasets should be directed to Thomas.Urup@regionh.dk.

## Ethics Statement

The study was performed according to the Declaration of Helsinki and Danish legislation and was approved by the local ethical committee (H-19054690). Exemption from obtaining informed consent to participate was granted by the ethical committee as patients were either deceased or fatally ill.

## Author Contributions

Patient inclusion and data collection was performed by KG. Data analysis was performed by AA, SM, and TU. Advanced statistical analyses was performed by IC. The first draft of the manuscript was written by AA. Review and editing were performed by all authors. All authors contributed to the article and approved the submitted version.

## Conflict of Interest

The authors declare that the research was conducted in the absence of any commercial or financial relationships that could be construed as a potential conflict of interest.
