# Supplementary material for: A Prognostic Model for Glioblastoma Patients Treated With Standard Therapy Based on a Prospective Cohort of Consecutive Non-Selected Patients From a Single Institution
Source: Front Oncol. 2021 Feb 25;11:597587. doi: 10.3389/fonc.2021.597587 (PMC7946965; doi:10.3389/fonc.2021.597587)
Supplement: Supplementary file 1 [file Table_1.docx]

| **Supplementary Table 1** Patient characteristics (*n* = 680) | |
| --- | --- |
| **Age (years), median (range)** | 60.2 (17.1-79.6) |
| **Gender, *n* (%)** |  |
| Female | 238 (35) |
| Male | 442 (65) |
| **ECOG performance status, *n* (%)** |  |
| 0 | 415 (62) |
| 1 | 220 (33) |
| 2 | 37 (5) |
| Missing | 8 |
| **Tumor size, median (range)** | 16 cm2 (0.25-61.5) |
| Missing | 345 |
| **Multifocal Disease, *n* (%)** |  |
| Yes | 83 (12) |
| No | 595 (88) |
| Missing | 2 |
| **Tumor localization, *n* (%)** |  |
| Frontal lobe | 139 (21) |
| Temporal lobe | 192 (28) |
| Parietal lobe | 76 (11) |
| Occipital lobe | 48 (7) |
| Overlapping | 161 (24) |
| Other | 62 (9) |
| Missing | 2 |
| **Secondary GBM^a^, *n* (%)** |  |
| Yes | 35 (5) |
| No | 645 (95) |
| Missing | 0 |
| **MGMT status, *n* (%)** |  |
| Methylated | 300 (53) |
| Unmethylated | 264 (47) |
| Missing | 116 |
| **IDH1 status, *n* (%)** |  |
| Mutation | 17 (4) |
| Wild-type | 400 (96) |
| Missing | 263 |
| **ATRX status, *n* (%)** |  |
| Positive | 135 (91) |
| Negative | 13 (9) |
| Missing | 532 |
| **EGFR status, *n* (%)** |  |
| Positive | 440 (82) |
| Negative | 95 (18) |
| Missing | 145 |
| **Tumor resection, *n* (%)** |  |
| Biopsy | 101 (15) |
| Resection | 579 (85) |
| **Surgically defined extent of resection, *n* (%)** |  |
| Partial resection | 254 (38) |
| Complete resection | 95 (62) |
| Missing | 7 |
| **MRI defined extent of resection^b^, *n* (%)** |  |
| Partial resection | 222 (63) |
| Complete resection | 130 (37) |
| Missing | 227 |
| **Corticosteroid use^c^,, *n* (%)** |  |
| Yes | 412 (61) |
| No | 262 (39) |
| Missing | 6 |
| **No. of adjvuvant temozolomide, *n* (%)** |  |
| **Median** | 4 |
| **Range** | 0 - 12 |
| 0 | 82 (12) |
| 1 | 49 (7) |
| 2 | 171 (25) |
| 3 | 34 (5) |
| 4 | 32 (5) |
| 5 | 73 (11) |
| 6 | 221 (33) |
| > 6 | 17 (2) |
| Missing | 1 |
| **Best clinical response, *n* (%)** |  |
| Response (complete response + progressive response) | 57 (8.8) |
| Stable disease | 320 (49.4) |
| Progressive disease | 271 (41.8) |
| Missing | 32 |
| **Follow-up duration (months), median (range)** | 80 (25.8-169.4) |
| *Abbreviations:* IDH1, isocitrate dehydrogenase 1;  MGMT, O6-methylguanine-DNA methyltransferase; ATRX, alpha thalassemia/mental retardation syndrome X-linked; EGFR, epidermal growth factor; MRI, magnetic resonance imaging.  ^a^Prior diagnosis with lower grade glioma or glioblastoma with IDH1 mutation.  ^b^ Assessed by MRI within 72 hours after surgery.  ^c^Prednisolone dose > 10 mg/day at initiation of concomitant treatment. | |

| **Supplementary Table 2** Univariate analysis: Response, Progression-free survival and Overall survival | | | |
| --- | --- | --- | --- |
| **Covariate** | **Response**  **(OR) [95% CI]** | **Progression-free survival**  **(HR) [95% CI]** | **Overall Survival**  **(HR) [95% CI]** |
| Resection |  |  |  |
| Biopsy *vs.* partial or complete resection | 0.312 [0.10 - 1.02] | 1.52 [1.20 - 1.89] | 1.47 [1.19 - 1.82] |
|  | p = 0.054 | p <0.001 | p <0 .001 |
| Surgically defined extent of resection |  |  |  |
| Biopsy *vs.* complete resection | 0.26 [0.079 - 0.88] | 1.63 [1.29 - 2.06] | 1.60 [1.27 - 2.02] |
| Biopsy *vs.* partial resection | 0.67 [0.37 - 1.20] | 1.36 [1.06 - 1.73] | 1.31 [1.04 - 1.67] |
|  | p = 0.059 | p < 0.001 | p < 0.001 |
| MRI defined extent of resection |  |  |  |
| Biopsy *vs.* gross total resection | 0.42 [0.11 - 1.60] | 1.62 [1.24 - 2.13] | 1.72 [1.31 - 2.26] |
| Biopsy *vs.* partial resection | 1.10 [0.48 - 2.56] | 1.28 [1.02 - 1.60] | 1.43 [1.14 - 1.80] |
|  | p = 0.3 | p = 0.002 | p < 0.001 |
| Age (per 10-year increase) | 0.72 [0.57 - 0.92] | 1.09 [1.02 - 1.17] | 1.20 [1.11 - 1.29] |
|  | p = 0.007 | p = 0.02 | p < 0.001 |
| Gender (female *vs.* male) | 0.84 [0.47 - 1.50] | 1.01 [0.86 - 1.19] | 1.06 [0.90 - 1.25] |
|  | p = 0.6 | p = 0.9 | p = 0.5 |
| Secondary glioblastoma^a^ *vs.* primary glioblastoma | 1.67 [0.48 - 5.83] | 0.47 [0.30 - 0.75] | 0.52 [0.33 - 0.81] |
|  | p = 0.4 | p < 0.001 | p = 0.004 |
| Multifocal *vs.* single lesion | 0.0 [0.00 - 0.00] | 1.54 [1.22 - 1.96] | 1.53 [1.21 - 1.93] |
|  | p = 1.0 | p < 0.001 | p < 0.001 |
| Frontal location *vs.* non-frontal location | 1.61 [0.87 - 2.97] | 0.85 [0.70 - 1.03] | 0.90 [0.74 - 1.09] |
|  | p = 0.13 | p = 0.10 | p = 0.3 |
| Temporal location *vs.* non-temporal location | 1.25 [0.66 - 2.34] | 0.99 [0.83 - 1.17] | 1.08 [0.91 - 1.28] |
|  | p = 0.5 | p = 0.9 | p = 0.4 |
| Parietal location *vs.* non-parietal location | 0.93 [0.39 - 2.26) | 1.08 [0.85 - 1.38) | 1.01 [0.79 - 1.29] |
|  | p = 0.9 | p = 0.5 | p = 0.9 |
| Occipital location *vs.* non-occipital location | 0.98 [0.34 - 2.85] | 1.03 [0.77 - 1.39) | 0.99 [0.73 - 1.33] |
|  | p = 1.0 | p = 0.8 | p = 0.9 |
| Tumor size (2-fold increase) | 1.34 [0.99 - 1.82] | 0.97 [0.90 - 1.08) | 1.00 [0.92 - 1.10] |
|  | p = 0.057 | p = 0.8 | p = 0.98 |
| Corticosteroid use^b^ (yes *vs.* no) | 0.63 [0.36 - 1.08] | 1.41 [1.20 - 1.65) | 1.56 [1.33 - 1.83] |
|  | p = 0.09 | p < 0.001 | p < 0.001 |
| ECOG performance status |  |  |  |
| 1 *vs.* 0 | 0.33 [1.52 - 0.71] | 1.42 [1.20 - 1.69] | 1.57 [1.32 - 1.85] |
| 2 *vs.* 0 | 0.80 [0.24 - 2.73] | 1.82 [1.28 - 2.59] | 2.95 [2.10 - 4.16] |
|  | p = 0.019 | p <0 .001 | p <0 .001 |
| MGMT unmethylated *vs.* methylated | 0.44 [0.23 - 0.83] | 1.39 [1.17 - 1.65] | 1.62 [1.36 - 1.92] |
|  | p = 0.01 | p < 0.001 | p < 0.001 |
| MGMT status classified by IHC, un-methylated vs. methylated | 0.39 [0.18 - 0.86] | 1.36 [1.08 - 1.65] | 1.65 [1.34 - 2.04] |
|  | p = 0.02 | p = 0.008 | p <0.001 |
| MGMT status classified by pyrosequencing un-methylated vs. methylated | 0.66 [0.19 - 2.25] | 1.49[1.10 - 2.02] | 1.58[1.12 - 2.14] |
|  | p = 0.5 | p = 0.01 | p =0.003 |
| Year of diagnosis |  |  |  |
| 2008-10 *vs.* 2005-7 | 0.54 [0.22 - 1.33] | 0.99 [0.76 - 1.30] | 1.05 [0.81 - 1.37] |
| 2011-12 *vs.* 2005-7 | 0.59 [0.27 - 1.30] | 1.06 [0.82 - 1.36] | 0.96 [0.75 - 1.23] |
| 2013-14 *vs.* 2005-7 | 0.55 [0.25 - 1.19] | 1.21[0.95 - 1.54] | 0.99 [0.78 - 1.27] |
| 2015-16 *vs.* 2005-7 | 0.43 [0.18 - 1.04] | 0.91 [0.70 - 1.18] | 0.96 [0.74 - 1.25] |
|  | p = 0.3 | p = 0.19 | p = 1.0 |
| *Abbreviations:* MRI, magnetic resonance imaging; MGMT, O6-methylguanine-DNA methyltransferase; IDH1, isocitrate dehydrogenase 1.  ^a^ Prior diagnosis with lower grade glioma or glioblastoma with IDH1 mutation.  ^b^Prednisolone dose > 10 mg/day at initiation of concomitant treatment. | | | |

| **Supplementary Table 3** Multiple imputations | | | | | |
| --- | --- | --- | --- | --- | --- |
|  | | EXP (Beta) | Lower CL | Upper CL | p-value |
| Outcome | Parameter |  |  |  |  |
| **Overall Survival (OS)** | MGMT, unmethylated | 1.54 | 1.30 | 1.83 | <0.0001 |
|  | Corticosteroid use^a^, yes | 1.42 | 1.20 | 1.68 | <0.0001 |
|  | Age, per 10-year increase | 1.20 | 1.11 | 1.29 | <0.0001 |
|  | Multifocal disease | 1.28 | 1.00 | 1.64 | 0.053 |
|  | ECOG Performance status 1 | 1.33 | 1.12 | 1.58 | 0.001 |
|  | ECOG Performance status 2 | 2.57 | 1.80 | 3.67 | <0.0001 |
|  | Resection | 0.83 | 0.66 | 1.66 | 0.1 |
| **Recurrence** | MGMT, unmethylated | 1.31 | 1.10 | 1.56 | 0.002 |
|  | Corticosteroid use, yes | 1.26 | 1.06 | 1.49 | 0.008 |
|  | Age, per 10-year increase | 1.07 | 1.00 | 1.16 | 0.06 |
|  | Multifocal disease | 1.32 | 1.03 | 1.70 | 0.03 |
|  | ECOG Performance status 1 | 1.27 | 1.07 | 1.52 | 0.008 |
|  | ECOG Performance status 2 | 1.53 | 1.07 | 2.20 | 0.02 |
|  | Resection | 0.78 | 0.62 | 0.99 | 0.04 |
| **Response** | MGMT, unmethylated | 0.71 | 0.55 | 0.94 | 0.02 |
|  | Corticosteroid use, yes | 1.11 | 0.87 | 1.41 | 0.4 |
|  | Age, per 10-year increase | 0.89 | 0.72 | 1.10 | 0.3 |
|  | Multifocal disease | 0.51 | 0.29 | 0.88 | 0.02 |
|  | ECOG Performance status 1 | 0.81 | 0.54 | 1.22 | 0.3 |
|  | ECOG Performance status 2 | 1.46 | 0.82 | 2.61 | 0.2 |
|  | Resection | 1.05 | 0.75 | 1.49 | 0.8 |
| *Abbreviations:* EXP (Beta), Hazards ratio for survival endpoints and odds ratio for response; CI, confidence level with 95% confidence interval; MGMT, O6-methylguanine-DNA methyltransferase.  ^a^Prednisolone dose > 10 mg/day at initiation of concomitant treatment. | | | | | |
